# TDP-43 dysfunction facilitates the pathological conversion of tau

**DOI:** 10.1186/s13024-026-00968-8

**Published:** 2026-07-04

**Authors:** Meghraj S. Baghel, Grace D. Burns, Margarita Tsapatsis, Aswathy Peethambaran Mallika, Anna Lourdes F. Cruz, Tianyu Cao, Xiaoke K. Chen, Isabel De La Rosa, Shaelyn R. Marx, Yingzhi Ye, Shuying Sun, Tong Li, Jonathan P. Ling, Philip C. Wong

**Affiliations:** 1 Department of Pathology, The Johns Hopkins University School of Medicine, 720 Rutland Avenue, Baltimore, MD 21205 USA; 2 Department of Physiology, The Johns Hopkins University School of Medicine, 720 Rutland Avenue, Baltimore, MD 21205 USA; 3 Department of Neuroscience, The Johns Hopkins University School of Medicine, 720 Rutland Avenue, Baltimore, MD 21205 USA; 4 Present address: Division of Neuroscience, National Institute on Aging, National Institutes of Health, Bethesda, MD 20814 USA

**Keywords:** TDP-43, Tau, Co-pathology, Vulnerable neuron, Alzheimer’s disease, ADRD, FTD, Neurodegeneration, Tauopathy, Caspase, Mouse model

## Abstract

TDP-43 proteinopathy coexists with tauopathy in a variety of neurodegenerative disorders, including Alzheimer’s Disease (AD) and AD related dementia (ADRD). While such co-pathology of TDP-43 is strongly associated with worsened neurodegeneration, the pathogenic mechanism underlying the exacerbated neuron loss remains elusive. Loss of TDP-43 splicing repression occurring during the early stage of neurodegenerative disease suggests that such loss could facilitate the pathological conversion of tau. Here, we report that TDP-43 loss-of-function (LOF) in forebrain neurons (*Tau4R*;* CaMKII-CreER; Tardbp*^*f/f*^ mice) exacerbates tauopathy-dependent brain atrophy is associated with vulnerable neurons sensitive to caspase 3-dependent cleavage of endogenous tau. We demonstrate that TDP-43 LOF in human iPSC-derived cortical neurons promotes TDP-43 dependent cryptic splicing which precedes caspase 3-mediated endoproteolysis of tau. Using a genetic approach to seed tauopathy in *CaMKII-CreER; Tardbp*^*f/f*^ mice by expressing a four-repeat microtubule binding domain of human tau, we show that the amount of tau seed correlates with caspase 3-dependent tau cleavage, accelerated tauopathy and the loss of vulnerable neurons deficient in TDP-43. Together, these results strongly support the view that TDP-43 dysfunction exacerbates tauopathy-dependent brain atrophy by promoting caspase 3-dependent endoproteolysis of tau, disclosing novel mechanistic insights and therapeutic targets for human tauopathies harboring the co-pathology of TDP-43.

**Supplementary Information:**

The online version contains supplementary material available at https://doi.org/10.1186/s13024-026-00968-8.

## Introduction

Alzheimer’s disease (AD), the most common age-related dementia, is associated with progressive loss of synapses, and eventually neurons [1]. While strong evidence supports a linear disease progression triggered by excessive amyloid-β (Aβ) in familial AD, multifactorial etiology has been hypothesized for sporadic late onset AD [2–13]. Recent studies have demonstrated that up to 75% of AD and AD-Related Dementia (ADRD) cases exhibit non-canonical pathologies, including TAR DNA/RNA binding protein (TDP-43) in limbic-predominant age-related TDP-43 encephalopathy (LATE) [14, 15], α-synuclein “Lewy bodies” and tau “pick bodies” [16–21]). TDP-43 pathology is observed in 40–60% of ADRD cases and is strongly associated with worsened neurodegeneration and cognition [16–18]). TDP-43 pathology is also observed in some cases of tauopathies including frontotemporal lobar degeneration (FTLD) [22], corticobasal degeneration (CBD) [23], primary age-related tauopathy (PART) [24] and progressive supranuclear palsy (PSP) [25]. Therefore, it is likely that the occurrence and interaction of these pathological factors will lead to worsened neurodegeneration and steeper cognitive decline.

TDP-43 is centrally associated with ALS-FTD [18] and regulates the repression of non-conserved cryptic exons [26]. Genetic, fluid biomarker and neuropathological findings from human studies support the notion that loss of TDP-43 splicing repression begins pre-symptomatically and drives neuron loss in ALS, FTLD-TDP and AD-TDP [27–41]. While the clinical significance of TDP-43 pathophysiology is undisputed, evidence as to how TDP-43 dysfunction accelerates neuron loss in mixed etiology dementia (MED) is currently lacking.

Human studies have reported co-localization and interaction of phosphorylated tau (p-tau) and TDP-43 positive inclusions in neurons of AD brains [42], but the exact nature of co-localization remains elusive. Furthermore, co-expression of tau and TDP-43 in animal models was shown to promote tau phosphorylation, suggesting synergism between tau and TDP-43 [43, 44]. Recent findings using a human autopsy cohort, tau biosensor line, and mutant TDP-43 mouse model support the idea that TDP-43 pathology correlates with more severe tauopathy [45].

We and others previously demonstrated that Aß plaque deposition is one necessary factor that facilitates the pathological conversion of endogenous tau in the presence of a human four-repeat domain (hTauRD) tau fragment serving as a seed to drive tauopathy and neuron loss [46], mimicking the pathogenic context within the AD brain. Likewise, intracerebral injection of AD-tau seeds facilitated Aß plaque-dependent misfolding of endogenous tau to drive neuron loss [47]. To test whether TDP-43 LOF facilitates the pathological conversion of endogenous tau, we took advantage of a mouse model in which loss of TDP-43 in forebrain neurons (*CaMKII-CreER; Tardbp*^*f/f*^ mice) leads to selective death of CA2/3 neurons accompanied by modest activation of caspase 3 [48] to model the early phase of human disease when TDP-43 dysfunction occurs [30]. To seed tauopathy, we employed two complementary approaches: (1) a crossbreeding strategy using *CaMKII-CreER; Tardbp*^*f/f*^ mice and *hTauRD* mice [46] to generate *CaMKII-CreER; Tardbp*^*f/f*^ mice expressing hTauRD in central neurons (*Tau4R*; *CaMKII-CreER; Tardbp*^*f/f*^ mice; Sup. Figure 1); and (2) intraparenchymal delivery of an adeno-associated viral vector expressing the *hTauRD* transgene (AAV-PhP.eB-hTauRD) in the hippocampus of *CaMKII-CreER; Tardbp*^*f/f*^ mice. Results from these complementary approaches converge on the idea that TDP-43 LOF promotes the pathological conversion of endogenous tau to drive tauopathy in vulnerable neurons. Furthermore, we explored the potential mechanism of pathological tau conversion and found that TDP-43 LOF facilitates the caspase 3-mediated endoproteolysis of tau in both mouse and human neurons. Together, our findings support the view that TDP-43 dysfunction promotes tauopathy and death of vulnerable neurons that are sensitive to caspase 3-dependent endoproteolysis of tau.

## Results

### Loss of TDP-43 function facilitates the pathological conversion of endogenous tau

**Fig. 1:**
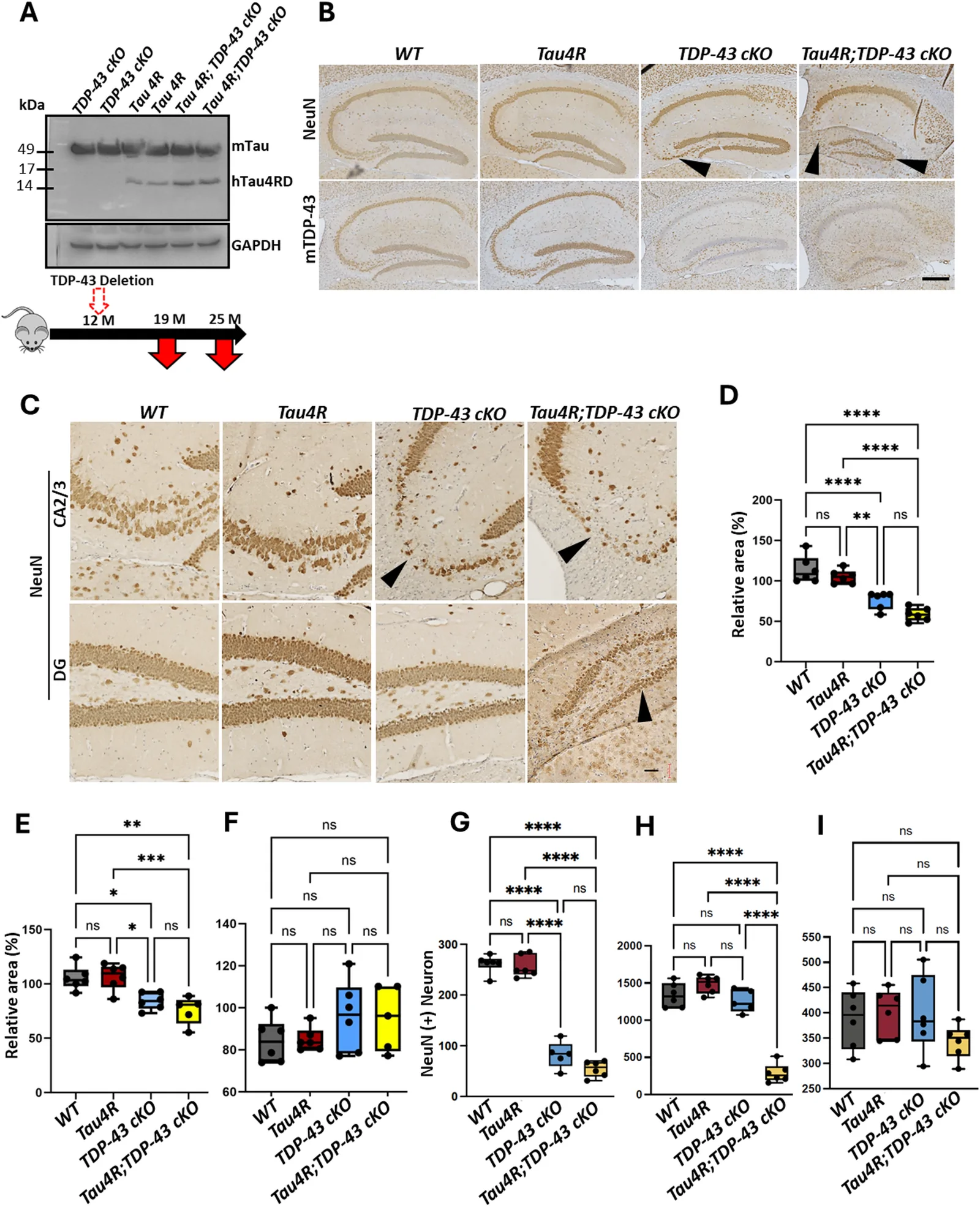
Loss of TDP-43 function accelerates neurodegeneration in *hTau4R* expressing mice.** (A)** Immunoblot using a KJ9A antibody which recognizes expression of the hTau4R fragment (~ 16 kDa) including endogenous mouse tau protein expression. **(B)** Immunohistochemical analysis of brains of 19-month-old *WT* (*n* = 6), *Tau4R* (*n* = 6), *TDP-43 cKO* (*n* = 5) and *Tau4R; TDP-43 cKO* mice (*n* = 6) using antibody specific to NeuN to detect neurons and antibody against mouse TDP-43 showing depletion of TDP-43 in the hippocampus (Arrow heads show the degenerated hippocampal neurons; scale bar, 200 μm). **(C)** Enlarged view of NeuN IHC, arrow heads indicating degenerated neurons in CA2/3 and DG subfield of hippocampus, scale bar, 20 μm). (**D-F**) Relative area measurements of hippocampus, cortex and cerebellum. **(G-I)** Neuron cell count of CA2/3 (panel G), DG (panel H), and CA1 (panel I) regions from 19-month-old cohort (ns: no significant difference; ****P < 0.0001). All graphs: median, IQR, min, max; one-way ANOVA

**Fig. 2:**
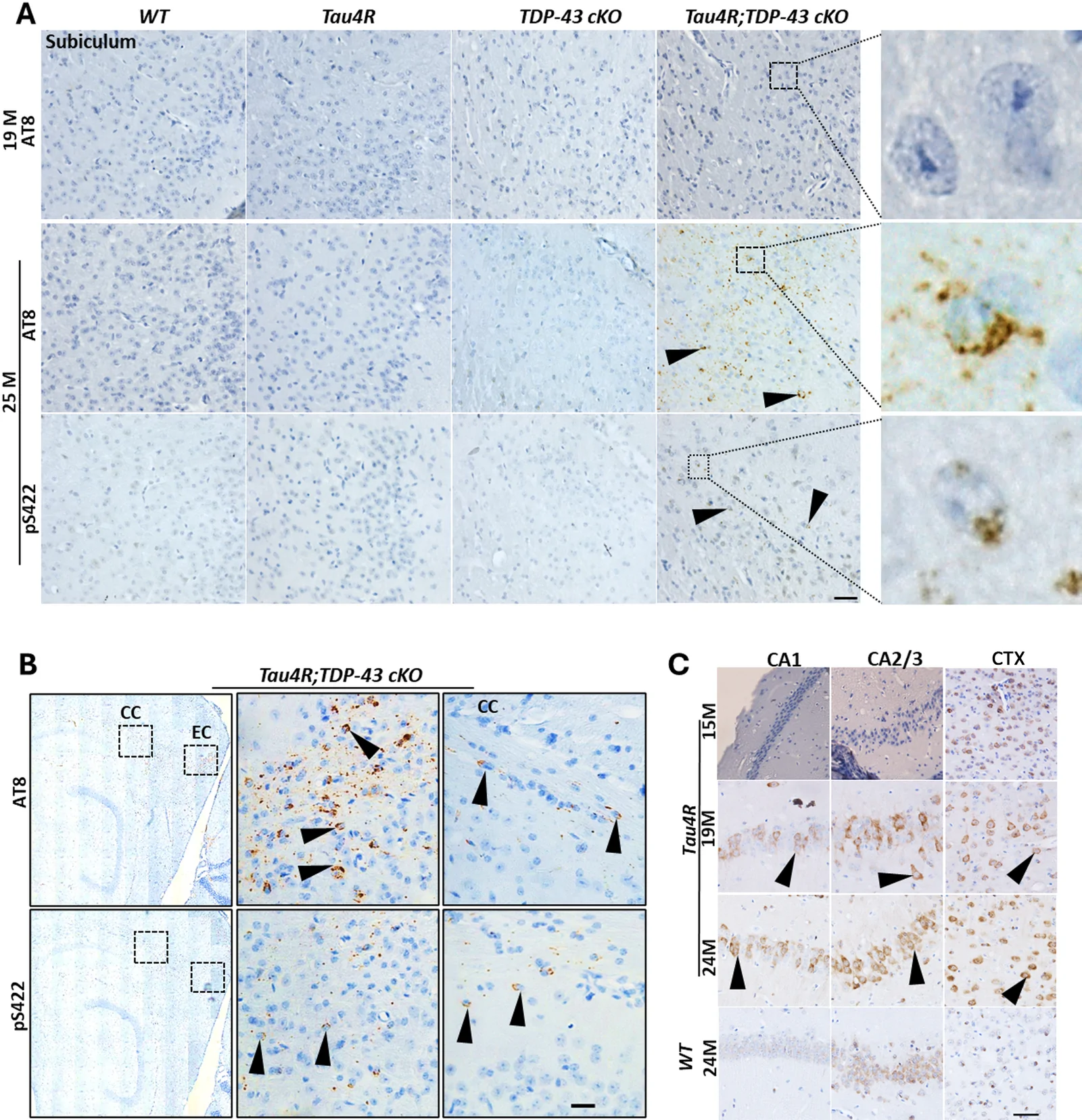
Loss of TDP-43 function facilitates pathological conversion of wild-type tau in *Tau4R; TDP-43 cKO* mice. **(A)** Immunohistochemical analysis of brain sections using antibodies specific to endogenous phosphorylated tau, AT8 (pSer202, pThr205, upper and middle panel) and pSer422 (lower panel). Arrow heads show immunoreactivity for pathological conversion of tau in *Tau4R; TDP-43 cKO* mice (scale bar, 40 μm followed by inset enlarged view right column). The sections were counterstained with hematoxylin (blue). **(B)** First occurrence of robust tau pathology in the entorhinal cortex (EC) including corpus callosum (first column) indicated by immunoreactivity with phosphorylated tau (pSer202, pThr205 & pSer422) in EC and CC of *Tau4R; TDP-43 cKO* mice (arrow heads indicates immunoreactivity in cells, scale bar, 20 μm). **(C)** Immunohistochemistry analysis showed an age-dependent increase in the accumulation of hTau4R fragment using antibody Tau4R repeat 7T4F in HP and CTX of *Tau4R* mice (aged to 15, 19 or 24 months of age), but not in 24-month-old *WT* mice (lower panel, scale bar, 50 μm)

Previously, we showed that a human tau seed (hTauRD) in the presence of Aß plaques facilitates the pathological conversion of endogenous tau to drive tauopathy and neuron loss [46]. We reasoned that such a tau seeding model could be used to determine whether TDP-43 LOF facilitates the pathological conversion of tau to drive tauopathy, leading to greater neuron loss, mimicking a human disease context with co-pathology of tau and TDP-43. To test this idea, we employed a crossbreeding strategy with *CaMKII-CreER; Tardbp*^*f/f*^ (*TDP-43 cKO*) and *Tau4R* mice to generate a cohort of *Tau4R; CaMKII-CreER; Tardbp*^*f/f*^ mice along with a set of littermate controls (Sup Fig. 1). We selectively deleted *Tardbp* in excitatory forebrain neurons at 12 months-of-age and analyzed these mice at various times up to 25 months. As expected, brain extracts from these mice subjected to immunoblot analysis confirmed the accumulation of the hTauRD (Tau4R) fragment (~ 16 kDa) in *Tau4R* and *Tau4R; CaMKII-CreER; Tardbp*^*f/f*^, but not control mice (Fig. 1A). As expected, there is no neuron loss in *Tau4R* transgenic mice (Fig. 2B-I) as compared to *WT*, which was demonstrated previously [46]. While depletion of TDP-43 led to selective loss of neurons in the CA2/3 subregion of the hippocampus of *CaMKII-CreER; Tardbp*^*f*/*f*^ mice (Fig. 1B-C, G) as expected [48], we observed a greater neuron loss including granule neurons in the dentate gyrus (DG) of *Tau4R; CaMKII-CreER; Tardbp*^*f*/*f*^ (Fig. 1B-C, H) but not control, mice. We also observed a trend, though non-significant, towards loss of CA1 neurons in *Tau4R; CaMKII-CreER; Tardbp*^*f*/*f*^ mice compared to littermate controls (Fig. 1I). Corroborating these observations, we found that while marked atrophy of the hippocampus and cortex was observed in *CaMKII-CreER; Tardbp*^*f*/*f*^ mice as expected, such atrophy was further exacerbated in *Tau4R; CaMKII-CreER; Tardbp*^*f*/*f*^ mice (Fig. 1D-E, Sup Fig. 2A-B). No significant difference was observed in the relative area of the cerebellum amongst all genotypes, which is expected because TDP-43 is not depleted in this region (Fig. 1F). These results are consistent with the idea that TDP-43 dysfunction occurring during the early stage of human disease promotes exacerbated neurodegeneration occurring in MEDs exhibiting the co-pathology of TDP-43 and tau.

Our findings that exacerbated neurodegeneration occurring in *Tau4R; CaMKII-CreER; Tardbp*^*f/f*^ mice and worsens as these mice age (by 25 months; Sup Fig. 2D) raised the question as to whether TDP-43 LOF can facilitate the pathological conversion of tau and drive age-dependent tauopathy. Immunohistochemical analysis of aged (25-month-old) *Tau4R; CaMKII-CreER; Tardbp*^*f/f*^ mice using two phosphorylated tau antibodies (AT8:Tau pSer202, pThr205 & pSer422) that bind outside the four-repeat domain revealed age-dependent pathological conversion of endogenous mouse tau, markedly in the hippocampal subiculum and entorhinal cortex (EC) (Fig. 2A-B; Sup Fig. 3) where tauopathy is thought to initiate in human disease. Pathological conversion of tau in these mice was detected at 25 months, but not 19 months, of age (Fig. 2A, upper row) accompanied by an age-dependent increase of Tau4R (Fig. 2C; Sup Fig. 4). Together, our results support the view that TDP-43 LOF sensitizes vulnerable neurons to facilitate the pathological conversion of endogenous tau.

### Elevated level of Tau4R seed exacerbates tauopathy in vulnerable neurons triggered by TDP-43 LOF

**Fig. 3:**
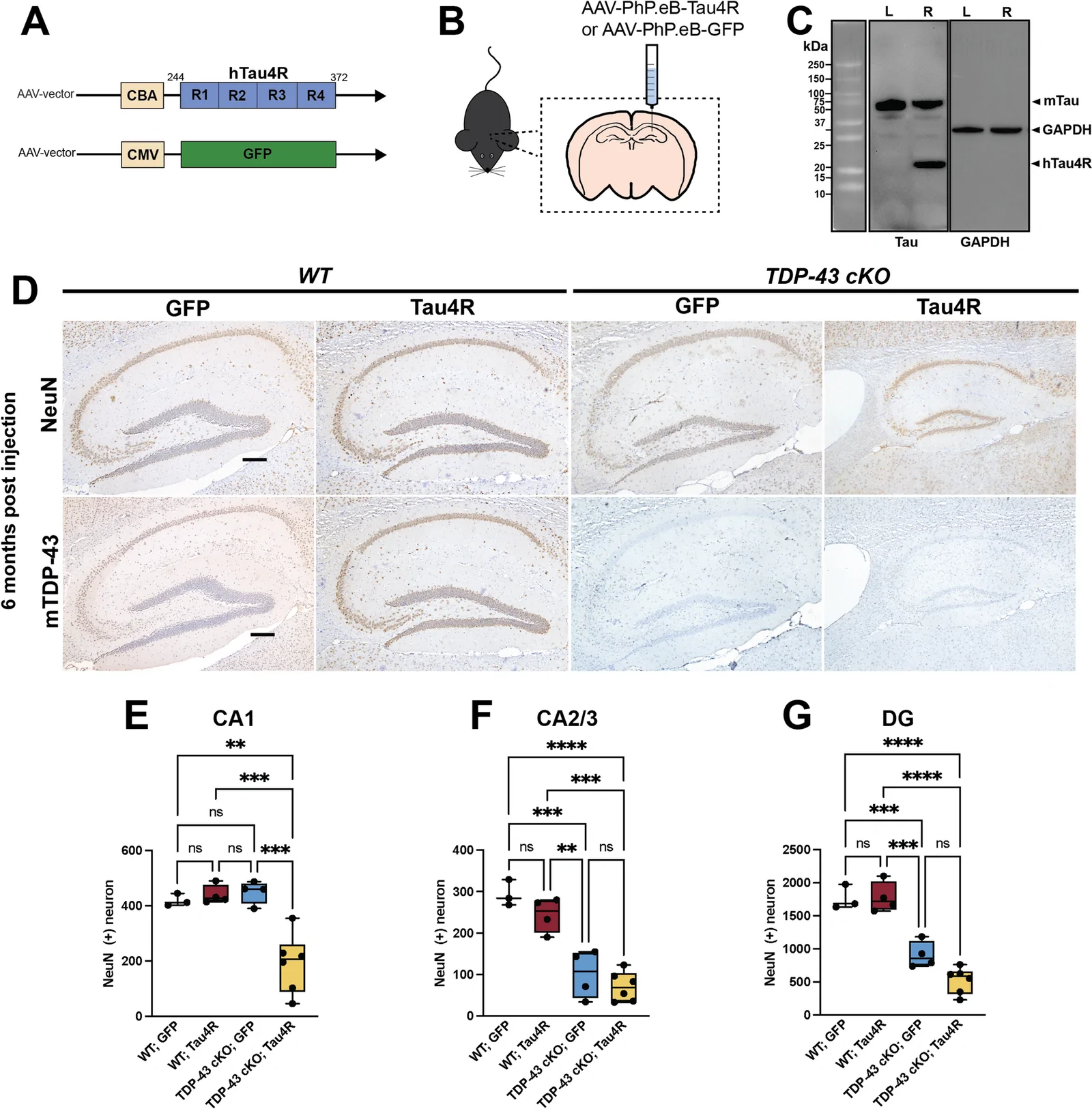
AAV delivery of *Tau4R* in brains of TDP-43-depleted mice exacerbates neurodegeneration. **(A)** Graphical illustration of the structure of expression vectors packaged into AAV-PhP.eB. The upper schematic diagram shows the construct with the four-repeat domain of human tau (hTau4R) downstream of the chicken β-actin (CBA) promoter. The lower one shows the control vector containing the construct encoding GFP downstream of the cytomegalovirus (CMV) promoter. **(B)** Schematic diagram showing the intraparenchymal injection of AAV-PhP.eB-hTau4R or AAV-PhP.eB-GFP into the right hippocampus. **(C)** Protein blot analysis using KJ9A antibody that recognizes the repeat domain of tau showed the presence of exogenous (∼16 kDa) hTau4R from hippocampal lysates of a mouse unilaterally 3–4 weeks post injected in the right hippocampi (R: right; L: left) with hTau4R. Note the accumulation of hTau4R is similar to that of endogenous tau in the right hemisphere. However, Tau-4R is undetectable in the left hemisphere. **(D)** Immunohistochemical analysis of brain sections from 18-month-old *WT* mice injected with AAV-PhP.eB-GFP (*n* = 3),* WT* mice injected with AAV-PhP.eB-Tau4R (*n* = 4), *TDP-43cKO* mice injected with AAV-PhP.eB-GFP (*n* = 4), and *TDP-43 cKO* mice injected with AAV-PhP.eB-Tau4R (*n* = 4) using antibody against NeuN or mouse TDP-43 (scale bar, 200 μm). **(E-G)** Neuron counts of CA1, CA2/3 and DG regions of the hippocampus (ns: no significant difference; CA1 **P = 0.003, ***P = 0.0007 (*WT; Tau4R* vs. *TDP-43 cKO; Tau4R*), ***P = 0.0005 (*TDP-43 cKO; GFP* vs. *TDP-43 cKO; Tau4R*); CA2/3 **P = 0.0019 (*WT; Tau4R* vs. *TDP-43 cKO; GFP*), ***P = 0.0003 (*WT; GFP* vs. *TDP-43 cKO; GFP*), ***P = 0.0001 (*WT; Tau4R* vs. *TDP-43 cKO; Tau4R*, ****P < 0.0001; DG ***P = 0.0005 (*WT; GFP* vs. *TDP-43 cKO; GFP*), ***P = 0.0002 (*WT; Tau4R* vs. *TDP-43 cKO; GFP*), ****P < 0.0001). All graphs: median, IQR, min, max; one-way ANOVA

Our observation that seeding low Tau4R levels in Tau4R transgenic mice facilitated tauopathy to include granular neuron loss in DG of *Tau4R; CaMKII-CreER; Tardbp*^*f/f*^ mice led us to ask whether higher levels of Tau4R expression in *CaMKII-CreER; Tardbp*^*f*/*f*^ mice could broaden the neuronal vulnerability in resistant neurons such as those within the CA1 of the hippocampus. To test this notion, we unilaterally injected AAV-PhP.eB expressing Tau4R or GFP as a control to seed tauopathy in the dorsal hippocampus of 12-month-old *CaMKII-CreER; Tardbp*^*f*/*f*^ mice to drive higher accumulation of Tau4R (Fig. 3A-B). Using an RNA probe designed to recognize an exon in the 5’-UTR of the AAV-PhP.eB vector, we first confirmed the presence of *Tau4R* RNA two months post injection in the ipsilateral side of the hippocampus and its adjacent region of the cortex (Sup Fig. 5A). Using an antibody recognizing Tau4R, we showed in the hippocampus Tau4R accumulated to a similar amount as endogenous tau (Fig. 3C), but to a markedly higher level found in *WT* mice injected with AAV-Tau4R as compared to *Tau4R* transgenic mice (Sup Fig. 5B-C).

As expected, six months post injection, *CaMKII-CreER; Tardbp*^*f*/*f*^ mice harboring the AAV-PHP.eB-GFP exhibited similar hippocampal atrophy (Fig. 3D) and CA2/3 vulnerability due to loss of TDP-43 (Fig. 3F). Intriguingly, because of increased tau seeding through AAV delivery, neuron loss in *CaMKII-CreER; Tardbp*^*f*/*f*^ mice harboring the AAV-PhP.eB-hTau4R extended beyond the CA2/3 and DG (Fig. 3F-G), as observed in *Tau4R; CaMKII-CreER; Tardbp*^*f/f*^ mice, to include those in the CA1 region (Fig. 3E).

**Fig. 4:**
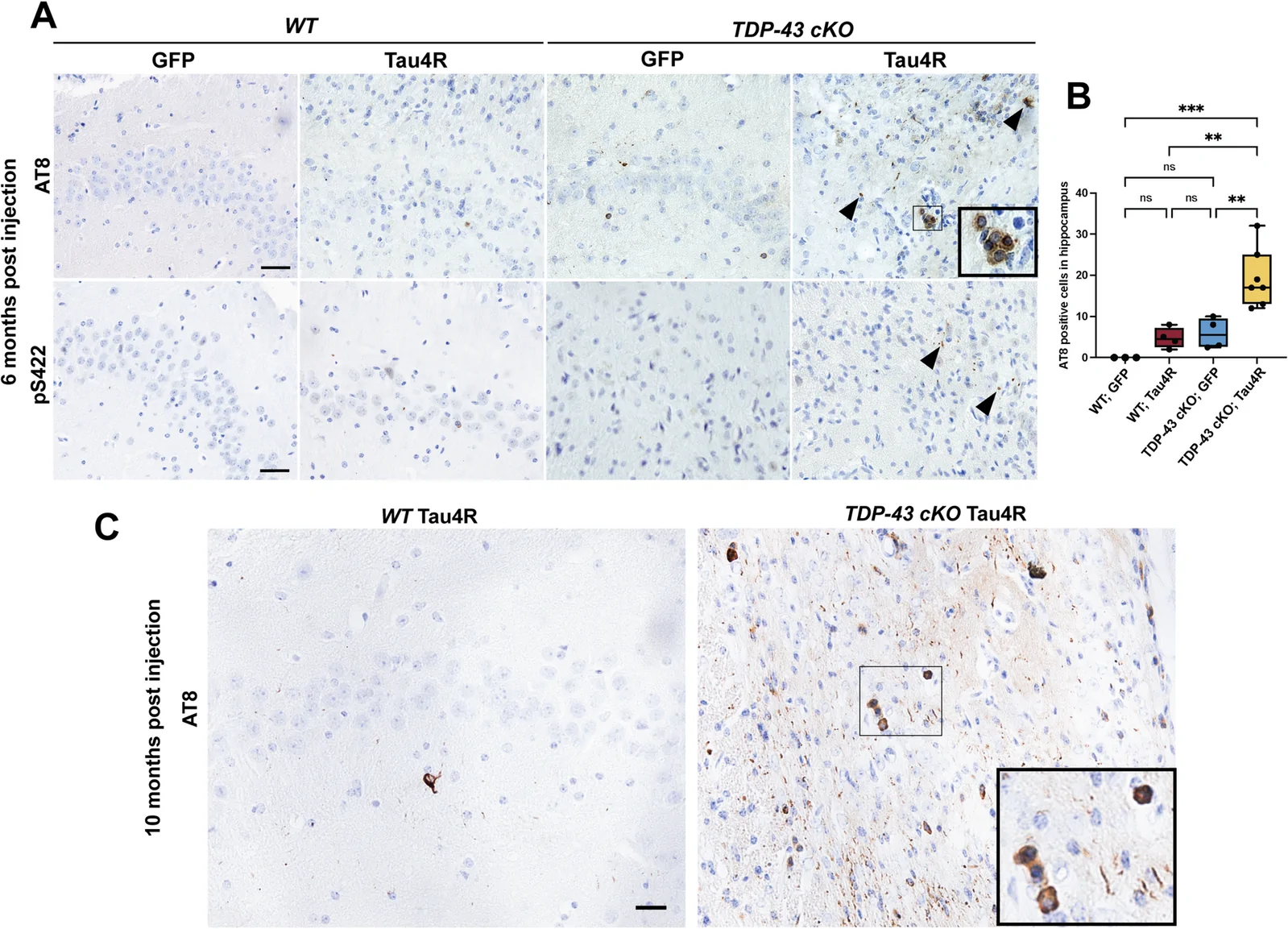
Loss of TDP-43 facilitates tauopathy through AAV delivery of a tau seed. **(A)** Immunohistochemical analysis and **(B)** quantification of brain sections of 18-month-old *WT* mice transduced with AAV-PhP.eB-GFP (*n* = 3), *WT* mice transduced with AAV-PhP.eB-Tau4R (*n* = 4), *TDP-43 cKO* mice transduced with AAV-PhP.eB-GFP (*n* = 4), and *TDP-43 cKO* mice transduced with AAV-PhP.eB-Tau4R (*n* = 4) using antibody against tau pSer202, pThr205 and pSer422 in the hippocampus (arrow heads depict the immunoreactivity of tau pSer202, pThr205 and pSer422; scale bar, 40 μm) (**P = 0.0021 (*WT; Tau4R* vs. *TDP-43 cKO*;*Tau4R*), **P = 0.0041 (*TDP-43 cKO; GFP* vs. *TDP-43 cKO; Tau4R*), ***P = 0.0004). Graph represents median, IQR, min, max; one-way ANOVA. **(C)** Immunohistochemical analysis of 22-month-old *WT* and *TDP-43 cKO* mice transduced with AAV-PhP.eB-Tau4R using antibody against tau pSer202, pThr205 (scale bar, 20 μm)

To determine whether higher levels of Tau4R could also accelerate the pathological conversion of tau, we examined the extent of tauopathy in AAV-PhP.eB-Tau4R-treated *CaMKII-CreER; Tardbp*^*f*/*f*^ mice using antibodies to pSer422 and pSer202, pThr205 (AT8). Six months post injection, we observed increased deposition of aggregated, hyperphosphorylated mouse (endogenous) tau as compared to that of littermate controls in the hippocampus of *CaMKII*-*CreER; Tardbp*^*f*/*f*^ mice injected with AAV-PhP.eB-Tau4R (Fig. 4A-B). This phenotype is further exacerbated 10 months post injection when *TDP-43 cKO* mice, as compared to *WT*, injected with Tau4R exhibit even greater phosphorylated tau burden (Fig. 4C). In the presence of greater levels of hippocampal Tau4R by AAV delivery, TDP-43 LOF accelerated the pathological conversion of endogenous tau to drive neuronal death to include those within the CA1 region.

Our complementary approaches establish that TDP-43 LOF can facilitate the pathological conversion of endogenous tau in the presence of a Tau4R seed leading to exacerbated neuronal loss, suggesting a pathogenic mechanism underlying tauopathies exhibiting the co-pathology of TDP-43.

### TDP-43 LOF facilitates caspase 3-mediated endoproteolysis of tau

**Fig. 5:**
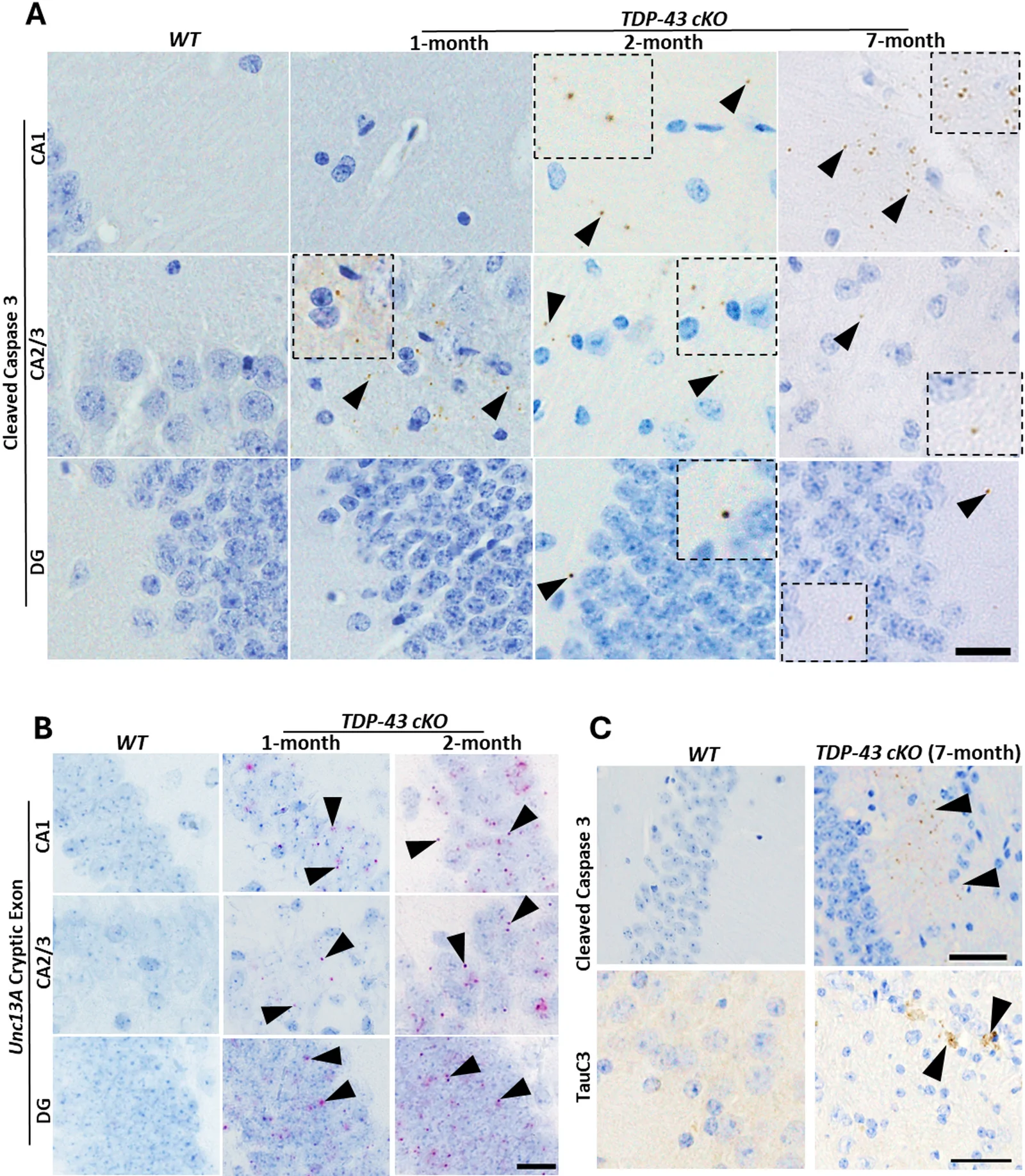
Loss of TDP-43 induces cryptic exon inclusion that precedes caspase-3 mediated cleavage of tau. **(A)** Cleaved caspase 3 immunohistochemical analyses in brain sections of *TDP-43 cKO* and *WT* (4 M) mice at different time points (TDP-43 depleted at 2 M of age, analyzed after 1, 2 M (columns 2 and 3); TDP-43 depleted at age of 12 M and analyzed after 7 months, right column). Enlarged views shown in insets of neurons (arrow heads) exhibiting cleaved caspase 3 immunoreactivity in dendritic fields of CA1, CA2/3 and DG subregions (scale bar, 20 μm). **(B)** BaseScope analysis of *Unc13a* cryptic exon in CA2/3, CA1 and DG in *WT* (4 M) and TDP-43 depleted (3, 4 M) mice (enlarged images with arrow heads point to BaseScope signals indicating cryptic RNA inclusions; scale bar, 50 μm). **(C)** Immunohistochemical analysis of *WT* (*n* = 3), and *TDP-43 cKO* (*n* = 6) mice depleted of TDP-43 at 12 months of age and analyzed at 19-months-old. Upper panel shows cleaved caspase 3 immunoreactivity in CA1, arrow heads indicate positive signal in right column (boxed area) (scale bar, 50 μm). Lower panel depicts the immunoreactivity of caspase 3 cleaved tau (TauC3), arrow heads show positive signal in right column (scale bar, 50 μm)

Since caspase 3 activation resulting in tau cleavage and aggregation has been linked to AD [49–54], we questioned whether this pathogenic mechanism was at play in our MED model. Previously, we showed that the deletion of TDP-43 in forebrain excitatory neurons led to selective loss of neurons in hippocampal CA2/3 accompanied by modest activation of caspase 3 [48]. Given that caspase activation dependent cleavage of tau precedes tau tangle formation [55], we asked whether cleavage of endogenous tau occurs in CA2/3, DG and CA1 neurons of *CaMKII-CreER; Tardbp*^*f/f*^ and *Tau4R; CaMKII-CreER; Tardbp*^*f/f*^ mice upon depletion of TDP-43. We observed selective activation of caspase 3 in the dendritic field of CA2/3 neurons, but not those in CA1 or DG, one month after the depletion of TDP-43 (Fig. 5A, second column); subsequently, caspase 3 was activated in CA1 and DG neurons two months after depletion of TDP-43 (Fig. 5A, third column) and accumulated over time (Fig. 5A, right column).

To determine whether TDP-43 LOF precedes caspase 3 activation, we assessed inclusion of a TDP-43 dependent cryptic exon within the mouse *Unc13a* pre-mRNA [56]. One month after depletion of TDP-43, we observed robust inclusion of this cryptic exon not only in CA2/3 neurons of *CaMKII-CreER; Tardbp*^*f/f*^ mice, but also those within CA1 and DG as assessed by a BaseScope probe that specifically identifies the *Unc13a* cryptic exon (Fig. 5B, middle column; Sup Fig. 9). These time-dependent regional observations strongly support the view that inclusion of cryptic exons precedes the activation of caspase 3 in *CaMKII-CreER; Tardbp*^*f/f*^ mice lacking TDP-43 in excitatory hippocampal neurons. Since caspase activation has been shown to cleave tau, an event that precedes tau tangle formation [55], we asked whether cleavage of endogenous tau occurs in CA2/3 neurons of *CaMKII-CreER; Tardbp*^*f/f*^ mice during aging. Importantly, TDP-43 LOF led to the activation of caspase 3 (cleaved caspase 3) dependent cleavage of endogenous tau (using antibodies, TauC3, recognizing the neoepitope exposed by caspase 3 at position D421 of tau) (Fig. 5C).

**Fig. 6:**
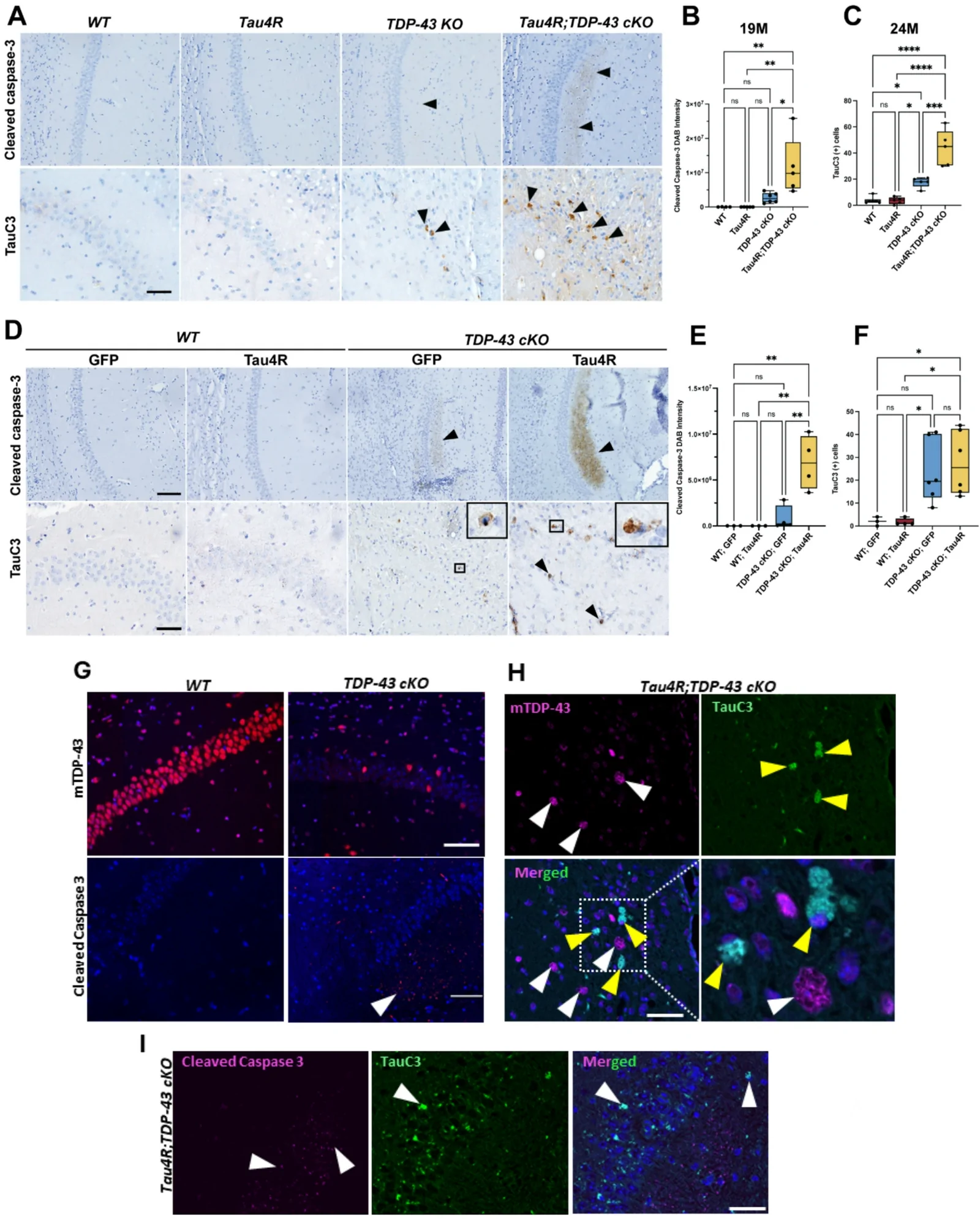
Depletion of TDP-43 facilitates caspase-3 activation and tau cleavage. **(A)** Immunohistochemical analysis and **(B-C)** quantification of brain sections of 19-month-old *WT* (*n* = 6), *Tau4R* (*n* = 6), *TDP-43 cKO* (*n* = 5) and *Tau4R; TDP-43 cKO* mice (*n* = 6) or 25-month-old *WT* (*n* = 3), *Tau4R* (*n* = 4), *TDP-43 *c*KO* (*n* = 5) and *Tau4R; TDP-43 cKO* mice (*n* = 4) in the CA1 region of the hippocampus using antibody against cleaved caspase 3 (scale bar, 50 μm) or caspase-cleaved tau (TauC3) in the CA2/3 region of the hippocampus (HP) (CA2/3) (scale bar, 50 μm). **(D)** Immunohistochemistry and **(E-F)** quantification of brain sections of 18-month-old *WT* mice transduced with AAV-PhP.eB-GFP (*n* = 3), *WT* mice transduced with AAV-PhP.eB-Tau4R (*n* = 4), *TDP-43 cKO* mice transduced with AAV-PhP.eB-GFP (*n* = 4), and *TDP-43 cKO* mice injected with Tau4R (*n* = 4) mice using antibody against cleaved caspase 3 in the CA1 region of the hippocampus (scale bar, 1000 μm) (**P = 0.0022 *WT; GFP vs. TDP-43 cKO; Tau4R*, **P = 0.0022 *WT; Tau4R vs. TDP-43 cKO; Tau4R*, **P = 0.0032 *TDP-43 cKO; GFP vs. TDP-43 cKO; Tau4R*) and caspase-cleaved tau (TauC3) in the CA2/3 region of the hippocampus (scale bar, 50 μm) (*P = 0.0282 *WT; GFP vs. TDP-43 cKO; Tau4R*, *P = 0.0403 *WT; Tau4R vs. TDP-43 cKO; GFP*, *P = 0.0146 *WT; Tau4R vs. TDP-43 cKO; Tau4R*). All graphs: median, IQR, min, max; one-way ANOVA. **(G)** Immunofluorescence analyses of brain sections of *TDP-43 cKO* or *WT* (4 M) mice, using antibody recognizing TDP-43 or cleaved caspase 3. Arrow heads indicate positive immunoreactivity for cleaved caspase 3 as puncta (lower panel, scale bar, 50 μm). **(H)** Representative images of double immunofluorescence staining using TDP-43 and TauC3 antibodies in *Tau4R; TDP-43 cKO* (25-month-old) mice, showing presence of neurons lacking nuclear TDP-43 with cleaved tau immunoreactivity as depicted by yellow arrow heads; in contrast, and the absence of TauC3 immunoreactivity is associated with presence of nuclear TDP-43 signal as indicated by white arrow heads (scale bar, 40 μm). **(I)** Representative images of double immunofluorescence staining using antibodies recognizing cleaved caspase 3 or TauC3 in *Tau4R; TDP-43 cKO* (25-month-old) mice, showing neurons with positive cleaved tau, but not activated caspase 3. immunoreactivity (indicated by arrow heads) (scale bar, 40 μm)

That only CA2/3 neurons are vulnerable to the loss of TDP-43 within the hippocampal circuit in *CaMKII-CreER; Tardbp*^*f/f*^ mice raised an interesting question as to whether other co-pathologic factors are required to activate caspase 3 above a certain threshold and sensitize other vulnerable neurons to tau cleavage. In the presence of a tau seed, depletion of TDP-43 sensitized granule neurons in DG. This finding led us to examine whether caspase 3 activation is elevated in *Tau4R; CaMKII-CreER; Tardbp*^*f/f*^ mice. As compared to *CaMKII*-*CreER; Tardbp*^*f*/*f*^ mice, a marked increase of activated caspase 3 was observed in granular neurons in DG of *Tau4R; CaMKII-CreER; Tardbp*^*f/f*^ mice (Sup Fig. 6B), suggesting that the vulnerability of hippocampal neurons depends on the level of activated caspase 3. Indeed, while activation of caspase 3 in the hippocampus occurred in *CaMKII*-*CreER; Tardbp*^*f*/*f*^ mice between 19 and 25 months-of-age, such caspase 3 activation was markedly accelerated in *Tau4R; CaMKII-CreER; Tardbp*^*f/f*^ mice (Fig. 6A-B, upper panel). This effect was age-dependent; 25-month-old mice exhibited enhanced caspase 3 activation, as compared to 19-month-old mice (Sup Fig. 6A).

To determine whether the caspase-mediated cleavage of tau is elevated in hippocampal neurons of *Tau4R; CaMKII-CreER; Tardbp*^*f/f*^ mice, we employed a TauC3 antibody specific to the cleavage site at D421. Immunohistochemical analysis revealed the presence of cleaved tau in an age dependent manner in hippocampal CA1, CA2/3, and cortex of *Tau4R*, *CaMKII-CreER; Tardbp*^*f*/*f*^ and *Tau4R; CaMKII-CreER; Tardbp*^*f/f*^ mice, but not in non-transgenic controls (Fig. 6A, lower panel; Sup Fig. 7C). As compared to *Tau4R* or *CaMKII-CreER; Tardbp*^*f*/*f*^ mice, a marked elevation of cleaved tau immunoreactivity is found in *Tau4R; CaMKII-CreER; Tardbp*^*f/f*^ mice (Fig. 6C; Sup Fig. 7C). The elevated level of cleaved tau was corroborated by immunoblotting using the TauC3 antibody (Sup Fig. 7B-C). Immunoreactivity was observed in CA2/3, cortex and CA1 (Fig. 6A, lower panel, Sup Fig. 6C & 7 A). We additionally confirmed the antibody specificity of TauC3 in *Tau4R; CaMKII-CreER; Tardbp*^*f/f*^ mice (Sup Fig. 7D, left two columns: negative primary and secondary antibody staining; right column; regular staining of TauC3). These results strongly support the idea that the vulnerability of neurons to pathological conversion of tau in *Tau4R; CaMKII-CreER; Tardbp*^*f/f*^ mice depends on the levels of activated caspase 3-cleaved tau, thus providing a potential pathogenic mechanism underlying MED harboring co-pathologies of tau and TDP-43.

Using our complementary approach, we observed accelerated caspase 3 activation in the CA1 dendritic field of *CaMKII-CreER; Tardbp*^*f*/*f*^ mice harboring AAV-PhP.eB-Tau4R. As compared to AAV-PhP.eB-GFP control mice, we observed marked increase in activation of caspase 3 in *CaMKII-CreER; Tardbp*^*f*/*f*^ mice harboring AAV-PhP.eB-Tau4R (Fig. 6D-E, upper panel). As expected, the degree of caspase activation correlated with caspase-cleaved tau in the hippocampus (Fig. 6D, F, lower panel). Our observations strongly support the notion that elevated levels of Tau4R promote greater activation of caspase 3-dependent cleavage of tau to accelerate the pathological conversion of endogenous tau to drive neuron loss.

Nuclear clearance of TDP-43 associates with the presence of cleaved tau in brains of a mouse model of MED. Given our observations that TDP-43 dysfunction facilitates caspase 3 activation-mediated cleavage of tau in both mouse and human models, a strong prediction is that, in neurons depleted of TDP-43, cleaved tau deposition can be observed in vivo. Indeed, while we found that depletion of TDP-43 restricted caspase 3 activation to the dendritic field of CA1 hippocampal neurons (Fig. 6G), cleaved tau was observed in TDP-43 depleted neuron cell body in the brains of *Tau4R; CaMKII-CreER; Tardbp*^*f/f*^ mice (Fig. 6H). Since activation of caspase 3 was not observed in cell body, we failed to demonstrate its co-colocalization with cleaved tau (Fig. 6I). 

## Depletion of TDP-43 dependent cryptic exon inclusion precedes caspase 3-mediated endoproteolysis of tau in human cortical neurons

**Fig. 7:**
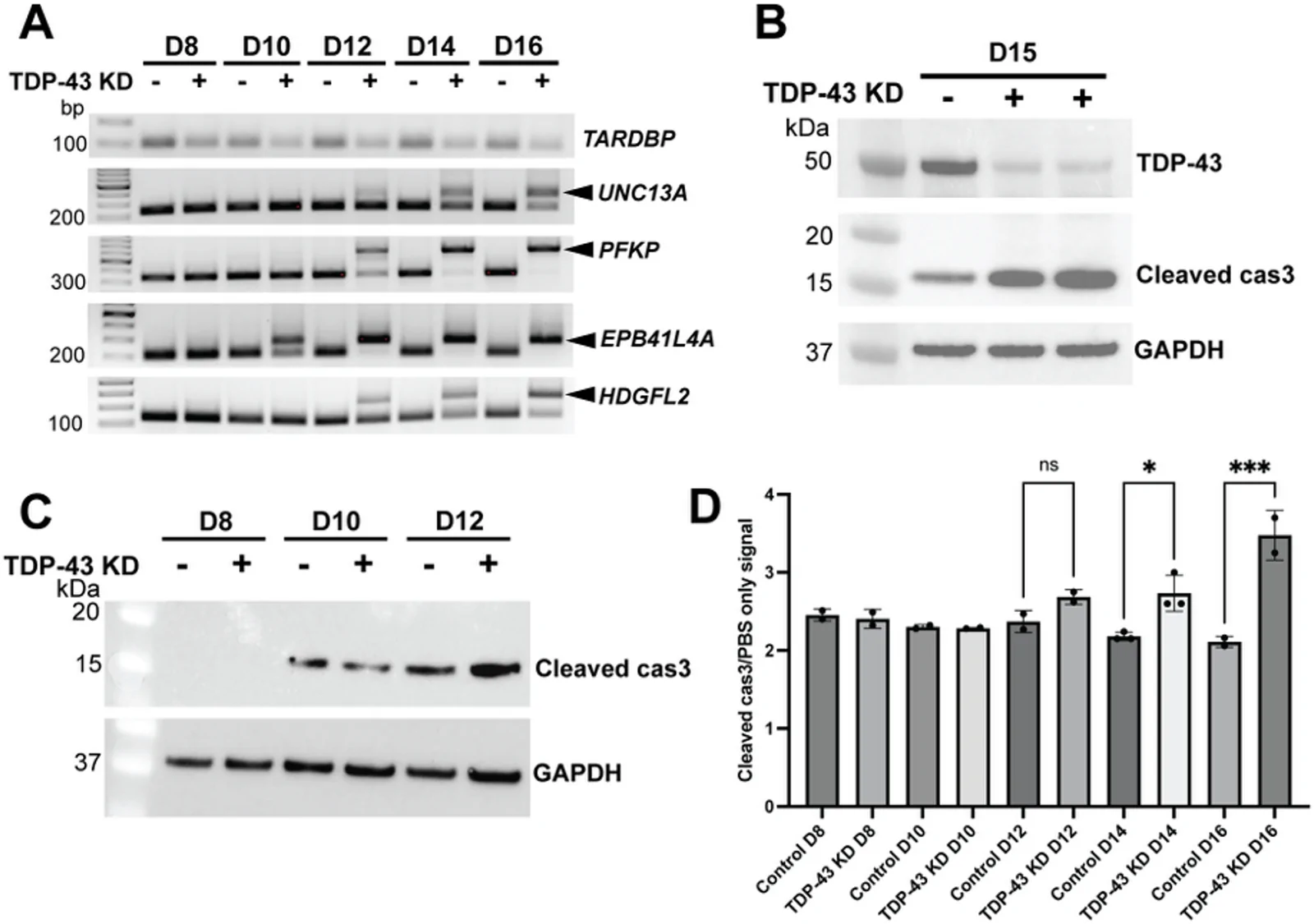
Depletion of TDP-43 leads to early cryptic exon inclusion and caspase-3 activation in human i3Neurons. **(A)** Reverse transcription-PCR analysis of TDP-43 cryptic exon targets at day 8, 10, 12, 14 or 16 of maturation of i3Neurons treated with a scramble lentivirus (control) or lentivirus targeting TDP-43; note that cryptic exon inclusion can begin as early as day 10. **(B)** Immunoblot showing knockdown of TDP-43 and robust cleaved caspase 3 at day 15 in TDP-43 KD i3Neurons. **(C)** Immunoblot showing levels of cleaved caspase-3 on days 8, 10, and 12. **(D)** MSD ELISA quantification of cleaved caspase-3 levels on days 8, 10, 12, 14, and 16 (*P = 0.038; ***P = 0.0005), *n* = 2 for each control, 2 or 3 for TDP-43 KD. Graph represents mean ± sd, one-way ANOVA

**Fig. 8:**
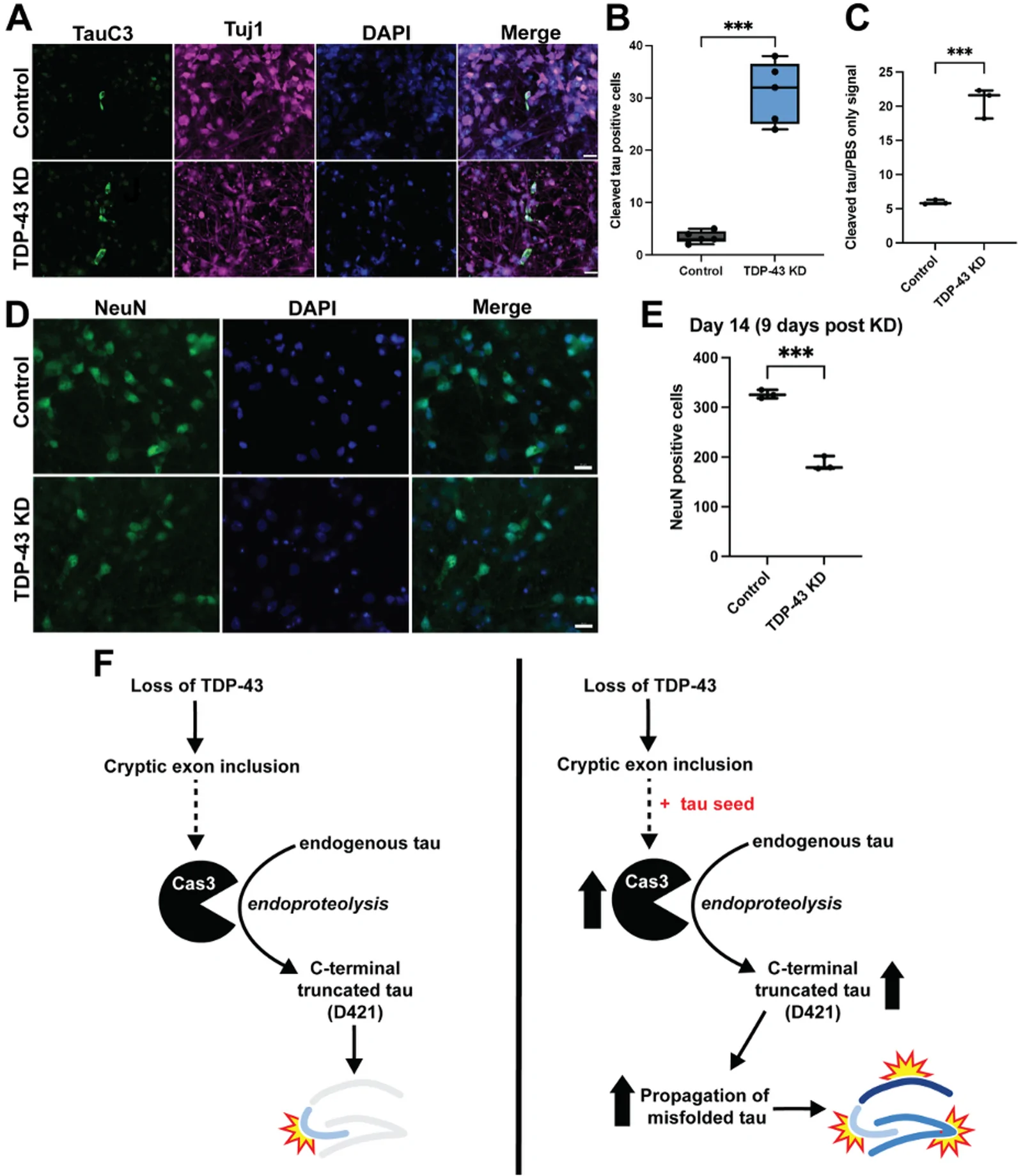
Loss of TDP-43 induces elevated cleaved tau levels and neuron loss. **(A)** Representative immunofluorescence images and **(B)** quantification of 14-day-old TDP-43 knockdown or control i3Neurons using antibodies against caspase-3-cleaved tau (TauC3) and ß-tubulin III showing increased cleaved tau and worsened axonal fragmentation in TDP-43 depleted cells (scale bar, 20 μm) (***P = 0.0005), *n* = 5. **(C)** Cleaved tau levels in control and TDP-43 KD i3Neurons on day 14 measured by MSD ELISA (***P = 0.0003), *n* = 3. **(D)** Representative immunofluorescent images and **(E)** quantification of NeuN positive cells in TDP-43 knockdown or control i3Neurons. (scale bar 20 μm) (***P = 0.0001), *n* = 3. All graphs: median, IQR, min, max; Unpaired t-test. **(F)** Model depicting that loss of TDP-43 splicing repression of cryptic exons precedes caspase 3-mediated cleavage of endogenous tau and eventual degeneration of CA2/3 neurons upon loss of TDP-43 in the mouse hippocampus. In the presence of a tau seed, elevated caspase 3-mediated cleavage of tau drives pathological tau aggregation and extends neuronal vulnerability to granule neurons of DG and CA1 neurons

To corroborate our finding that TDP-43 cryptic exon inclusion occurs prior to caspase 3 activation in the human context, we depleted TDP-43 through exposure of lentivirus expressing an shRNA targeting TDP-43 (shTDP-43) in human iPSC-derived i3Neurons [57]. As assessed by RT-PCR, immunoblot and immunofluorescence analysis, we observed efficient knockdown of TDP-43 as compared to the non-targeting, scrambled control (Fig. 7A, B; Sup Fig. 9A). Intriguingly, for some of the tested cryptic exons, we observed cryptic exon (*EPB41L4A*) inclusion first in day 10, by day 12 and onwards, most cryptic exons can be observed in TDP-43 KD neurons (Fig. 7A; Table 1). To correlate with caspase 3 activation in a time-dependent manner, while we found by day 8, there is no caspase 3 activation in TDP-43 negative or positive neurons (Fig. 7C, cleaved caspase 3, 17 kDa), but by day 10, we observed basal levels of caspase 3 activation in both TDP-43 positive and negative neurons (as assessed by immunoblotting, Fig. 7C; and MSD assay Fig. 7D). We thus interpret this to indicate that low levels of caspase 3 could be detected under in-vitro conditions or during normal aging neurons. Strikingly, by day 12, we started to observe an upward trend of activated caspase 3 levels in TDP-43 KD neurons as determined by immunoblotting and MSD assay (Fig. 7C-D). Further, by day 14 and onwards, levels of activated caspase 3 were significantly different between TDP-43 negative, as compared to positive, neurons (Fig. 7B, D). And such difference is preceded by cryptic exon inclusion reaching their highest level at day 12. Thus, this time-dependent study in i3Neurons strongly suggests that cryptic exon inclusion precedes caspase 3 activation occurring in TDP-43 KD neurons. These observations in i3Neurons are consistent with our findings in mice and strongly support the notion that inclusion of TDP-43 dependent cryptic exons precedes caspase activation. As expected, we showed that loss of TDP-43 induces marked cleavage of endogenous tau (Fig. 8A-C). Since caspase 3 activation precedes tau cleavage, our observation of cleaved caspase 3 and cleaved tau colocalization (Sup Fig. 8C) confirms that cleaved tau eventually accumulates upon caspase 3 activation, leading to neuron loss (Fig. 8D-E). This observation is consistent with our mouse models where TDP-43 depletion drives caspase 3 activation-mediated cleavage of tau to amplify tauopathy.

Together, our data strongly supports the notion that TDP-43 dysfunction facilitates pathological conversion of endogenous tau in the presence of Tau4R seed, leading to exacerbated neuron loss. In the mouse hippocampus, TDP-43 LOF and elevated level of Tau4R seed accelerates pathological conversion of tau and increased neuronal vulnerability to cell death. Thus, our findings offer a potential pathogenic mechanism underlying accelerated neuron loss occurring in tauopathies with co-pathology of TDP-43: TDP-43 dysfunction promotes caspase 3-mediated cleavage of tau to drive tauopathy (Fig. 8F).

## Methods

### Mouse models

We used the conditional knockout mouse model of TDP-43 by generating *CaMKIIα-Cre*^*ER*^;*Tardbp*^*f/f*^ (*TDP-43 cKO*) mice with loxP sites flanking *Tardbp* exon 3 [48], creating a tamoxifen-induced recombination in excitatory forebrain neurons in adult mice. Through a two-stage breeding strategy (Supp Fig. 1), *CaMKIIα-Cre*^*ER*^;*Tardbp*^*f/f*^ mice bred with *tTA; Tau4R* (Tau4R) line generated previously [46] and a cohort of *tTA; Tau4R; CaMKIIα-Cre*^*ER*^;*Tardbp*^*f/f*^ (*Tau4R; TDP-43 cKO*) mice on a C57BL/6J background, as well as littermate controls. Animals were genotyped at weaning, and mice were subsequently housed with one littermate of each genotype (5 mice/cage) when possible. Oral administration of tamoxifen citrate through diet was done in all animals (Harlan Teklad) at an average amount of 40 mg/kg/day for a 4-week period, beginning at 12 months-of-age. Studies in *Tau4R; TDP-43 cKO* mice and littermate controls were carried out in brain tissues from 19- or 25-month-old mice.

For the AAV-PhP.eB-Tau4R/GFP injection model, we deleted TDP-43 at 11 months-of-age through the administration of tamoxifen feed. Mice were returned to normal feed as recovery for 2 weeks and then stereotaxic surgery was performed.

### Ethics declaration

All the animal experiments were conducted as per the regulations of the Animal Care and Use Committee at Johns Hopkins University School of Medicine in accordance with the laws of the State of Maryland and the United States of America and employing standard protocols. The protocol number is MO24M137, which was approved on 05/07/2024.

### Cell culture and transduction

The i3Neuron iPSC line was adapted from Michael Ward’s lab. iPSCs were cultured in Essential 8 media (Gibco, A1517001) on plates coated with Geltrex (Gibco, A1413301). iPSCs were differentiated into i3Neurons as described before [57]. Lentivirus containing scrambled short hairpin RNA as non-targeting control (shScramble) or targeting TDP-43 (shTDP-43) as used before [58], was transduced on day 5 after differentiation. Thereafter, neurons were harvested on day 8 to 16 or day 14 and 15 after differentiation, respectively, for cryptic exon analysis or immunoblot and immunostaining TDP-43, cleaved caspase 3 and cleaved tau.

### RT-PCR analysis

RNA was extracted from samples using the Monarch^®^ Total RNA Miniprep Kit (New England Biolabs, T2010S) and cDNA was synthesized using the ProtoScript II First Strand cDNA Synthesis Kit (New England Biolabs, E6560L). Target sequences were amplified using a modified touchdown PCR protocol [59, 60], primer pairs targeting wildtype with or without cryptic sequences (see table below) and Phusion Plus Green PCR Master Mix (Thermo Scientific, F632L). Upon cryptic exon inclusion, a larger band (CE) is expected. Amplified products were then separated by 1.5% agarose gel electrophoresis and visualized by ethidium bromide staining.

**Table 1:** Primer sequences for RT-PCR analysis of mRNAs for human *TARDBP* or its cryptic target genes

Target	Forward primer (5’-> 3’)	Reverse primer (5’ -> 3’)	Expected band sizes (bp)
*TARDBP*	AATTCTGCATGCCCCAGA	GAAGCATCTGTCTCATCCATTTT	96
*UNC13A*	CAACCTGGACAAGCGAACTG	GGGCTGTCTCATCGTAGTAAAC	WT: 127, CE: 355
*PFKP*	AAGTTCCTGGAGCACCTCTC	CACACACAGGTTGGTGATGC	WT: 330, CE: 593
*EPB41L4A*	TGTGACAGAAGCCATCAGACG	GCCCTGAAGGACATCTTGCT	WT: 95, CE: 270
*HDGFL2*	CAGCGACCAGGACTTCACAC	GAATCGGCCTTGGAGTCGGA	WT: 123, CE: 261

### Intraparenchymal stereotaxic injection

Mice were deeply anesthetized with isoflurane and fixed in a stereotaxic frame. The scalp skin of the animal was swabbed with povidone iodine and the skull was exposed via an incision. The skull was cleaned with hydrogen peroxide to allow for a craniotomy to be made above the right hippocampus using coordinates for dorsal hippocampus (2 mm frontal to lambda, 2 mm lateral to midline, and 1.5 mm depth) from the skull surface at the rate of 1µL/min as standardized before [61]. Unilateral injections were performed using a syringe (Hamilton) containing 5 µL of 2 × 10^13^ vg/mL AAV-PhP.eB-GFP or Tau4R. Upon completion, the needle was slowly withdrawn for 3 min. The skin was stapled and covered with 2% chlorohexidine to heal the incision.

### Immunoblotting

For protein blot analysis of cultured neurons, total protein was extracted with RIPA buffer (10 mM Tris-Cl (pH 8.0), 1 mM EDTA, 0.5 mM EGTA, 1% Triton X-100, 0.1% sodium deoxycholate, 0.1% SDS and 140 mM NaCl) containing 1X protease inhibitor cocktail (Roche, Indianapolis, IN). Samples were sonicated at 20 kHz for three five second pulses. Protein concentrations were determined by the BCA assay (Pierce Chemical Co., Rockford, IL) and equal amounts of protein lysates (∼20 µg per lane) were resolved on 4–12% Bis-Tris SDS–PAGE gels and then transferred to polyvinylidene difluoride membranes (Invitrogen, Carlsbad, CA). After blocking with 5% skimmed milk, the membranes were probed with the following antibodies: anti-TDP-43 N-terminus (1:2,000; 10782-2-AP; ProteinTech) and cleaved caspase 3 (1:1000; 5A1E; Cell Signaling Technology). Immunoblots were developed using enhanced chemiluminescence method (Millipore Corp., MA).

Total protein from the mouse hippocampus was extracted by homogenization in RIPA buffer. For mice injected with AAV, 3–4 weeks post-injection left, and right hippocampi were dissected separately and total protein was extracted. For the analysis of cleaved tau levels, total protein was extracted from cortexes of 19 months old mice. Protein concentrations were determined, and equal amounts of protein lysates (∼20 µg per lane) were resolved, transferred to polyvinylidene difluoride membranes, and probed with the following antibodies: anti-human tau polyclonal antibody KJ9A (1:5,000; A0024, Dako Cooperation, Carpinteria, CA), TauC3 (1:1000; AHB0061, Invitrogen) and rabbit anti-GAPDH antibody (1:5,000; G9545, Sigma). Immunoblots were developed using enhanced chemiluminescence method (Millipore Corp., MA).

### Immunofluorescence staining

For immunofluorescent staining of i3Neurons, cells were fixed with an equal volume of 8% PFA to cell culture medium and permeabilized with 0.1% Triton X. Blocking buffer (10% normal goat serum in PBS) was applied for an hour followed by primary antibodies overnight at 4 °C. Secondary antibodies (Alexa Fluor 488, Alexa Fluor 594, Alexa Fluor 647) were added, and cells were stored in PBS for imaging. For quantification of cleaved tau and NeuN positive cells, 10 random captured images were used to quantify manually per biological replicate.

The following primary antibodies were used: antibody against TDP-43 C-terminus (1:1000; 12892-1-AP; ProteinTech), MAP2 (1:1000; 188004; Synaptic Systems), cleaved caspase 3 (1:1000; D3E9; Cell Signaling Technology), Tuj1 (1:500; ab18207; Abcam), and cleaved tau (TauC3) (1:400; AHB0061, Invitrogen). The Zeiss Apotome Inverted Fluorescence Microscope (Zeiss, Germany) and the Zeiss LSM 800 AiryScan.2 were used for imaging.

To perform immunofluorescence staining on tissue, the paraffin brain sections were deparaffinized and antigen retrieval was performed using 10mM sodium citrate buffer to expose the epitope to the antibodies. Nonspecific binding of antibodies was eliminated by incubating with blocking buffer (1.5% normal goat serum in PBS with 0.1% Triton-X) for 1 h. After blocking, the primary antibodies against Tau4R (E7T4F) (1:1000; 79327, Cell Signaling Technology), against cleaved caspase 3 (1:2000; D3E9; Cell Signaling Technology) and TDP-43 (TDP-43 N-terminus (1:1,000; 10782-2-AP, ProteinTech). For double immunofluorescence sections were incubated with antibody against TDP-43 and cleaved caspase 3 (1:2000; D3E9; Cell Signaling Technology) and cleaved tau (TauC3) (1:400; AHB0061, Invitrogen) or cleaved caspase 3 and cleaved tau antibodies were incubated together overnight in humid chambers. Unbound antibodies were washed out and incubated with respective secondary conjugated fluorophores (Alexa Fluor 488 & Alexa Fluor 594). The Zeiss Apotome Inverted Fluorescence Microscope (Zeiss, Germany) was used for imaging.

### Histology and immunohistochemistry

*Tau4R; TDP-43 cKO* mice and their age matched littermates were sacrificed at 19 or 25 months of age for analysis. AAV-mediated Tau4R mice were sacrificed 6 months post AAV-Tau4R injection. Mice were anesthetized with isoflurane and transcardially perfused with ice-cold PBS. Brains were dissected, postfixed in 4% PFA for 24 h, embedded in paraffin and then sectioned with a microtome into 10 μm thick sagittal sections.

For immunohistochemistry, the slides were deparaffinized and antigen retrieval was performed using 10mM sodium citrate buffer to efficiently expose the epitope to the antibodies. Nonspecific binding of antibodies was eliminated by incubating with blocking buffer (1.5% normal goat serum in PBS with 0.1% Triton-X) for 1 h. Slides were then incubated in 0.3% H_2_O_2_ for 30 min to quench endogenous peroxidase. Primary antibodies were prepared in blocking buffer and applied overnight at room temperature followed by incubation with biotinylated secondary antibody for one hour. Peroxidase labeled ABC reagent (Vector Laboratories) was applied for 30 min followed by signal development using 3,3 diaminobenzidine (DAB) (Vector Laboratories). Slides were counterstained with hematoxylin, dehydrated, cleared and mounted.

The following primary antibodies were used: antibody against NeuN (1:1,000; MAB377, Merck); antibody against TDP-43 N-terminus (1:1,000; 10782-2-AP, ProteinTech); phosphorylated S422 of tau (1:1,000; 44764G, Invitrogen, Carlsbad, CA); AT8 (pSer202, pThr205) (1:1,000; MN1020, Invitrogen); antibody against Tau4R (E7T4F) (1:1000; 79327, Cell Signaling Technology); antibody against cleaved Caspase 3 (1:2000; D3E9; Cell Signaling Technology); antibody against cleaved tau (TauC3) (1:200; AHB0061, Invitrogen).

The areas of hippocampus, cortex, and cerebellum in sagittal sections at 2 mm from the midline of the brains were measured using ImageJ. At three different levels, we combined (“stitched”) images from 3 sections to generate a composite representing each sagittal section (Sup Fig. 2). For each mouse, the ImageJ program was used to quantify the relative area by defined boundaries to include hippocampus, cortex, and cerebellum for each of the three composites; averaged values for each of the brain regions were determined. The numbers of neurons in the CA1, CA2/3, and DG regions were counted using ImageJ. These analyses were quantified using three separate individuals in a blinded fashion (i.e., genotypes were not disclosed to the investigators) and values were averaged for statistical analysis.

ImageJ was used to quantify cleaved caspase 3 DAB staining. A minimum of 2–3 sections for each mouse were used to quantify DAB staining. Images were color deconvoluted, and detection threshold was set manually by comparison to the original signal. The measured relative DAB intensity values were plotted.

For AT8 quantification, we manually counted the AT8 positive cells in hippocampal sections similar to our NeuN quantification. We used 2–3 slices from each mouse to quantify the AT8 positive cells then averaged in different groups.

For TauC3 quantification, we manually counted the TauC3 occurring in cell body of positive cells in hippocampal sections. We used 2–3 sections from each mouse to quantify the TauC3 positive cells for each genotype.

### Cleaved caspase-3 or cleaved tau meso scale discovery assay

Each well on the Meso Scale Discovery (MSD) MULTI-ARRAY standard plate was coated with 40 µL of cleaved caspase-3 antibody (5A1E; Cell Signaling Technology) or cleaved tau antibody (AHB0061, Invitrogen) at 2 µg/mL and incubated overnight at 4 °C. Plates were washed three times with 150 µL PBS-T (0.05% Tween) and were blocked with 150 µL blocking buffer (5% BSA in PBS-T) at room temperature for one hour with shaking at 700 rpm. Samples were added to the plate in duplicate following three more washes with 150 µL PBS-T. Samples were incubated overnight at 4 °C with shaking at 700 rpm. Following sample incubation, plates were washed three times with 150 µL PBS-T. Detection antibodies were then added at 10 ug/mL. For the cleaved caspase-3 assay, the detection antibodies used were anti-caspase-3/P17/P19 (66470-2-Ig, ProteinTech) and anti-mouse SULFO-TAG labeled (R32AC-5, MSD). For the cleaved tau assay, the detection antibody (anti-Tau, 316–355 clone 77G7, Biolegend) was SULFO-TAG conjugated (conjugation ratio: 12.6). The detection antibody was incubated at room temperature for two hours with shaking at 700 rpm. The plate was then washed three more times with 150 µL PBS-T, and then 150 µL MSD GOLD Read Buffer A was added per well. The plate was immediately read on the MESO QuickPlex SQ 120MM instrument.

### BaseScope-ISH and co-detection assay

BaseScope in situ hybridization was performed using BaseScope Detection Reagent v2-RED Assay Kit (Cat#323900, Advanced Cell Diagnostics, Inc.), following the manufacturer’s instructions. We used 3ZZ probes targeting an exon within the 5’-UTR of the AAV-PHP.eB vector (BA-CAG-promoter-O1-3zz-st-C1, Cat#1259091-C1, ACD Bio). A custom BaseScope probe (BA-Mm-*Unc13a*-O1-2EJ-C, Cat#1182491-C1, ACDBio) was used to detect the transcripts containing cryptic exon splice sites in *Unc13a*. To ensure RNA integrity, both positive (BA-Mm-Ppib, Cat#701071) and negative (BA-DapB, Cat#701011) control probes were used. Briefly, 10 μm tissue sections were deparaffinized and pre-treated with hydrogen peroxide, target retrieval buffer and protease IV and then hybridized with the target probes in a HybEZII oven (Advanced Cell Diagnostics, Inc.) for 2 h at 40 °C. The signals were amplified, and the sections were counterstained with hematoxylin. Images showing RNA puncta for each cryptic exon were captured using a Zeiss Apotome Inverted Brightfield Microscope (Zeiss, Germany).

To enable the simultaneous detection of transcripts containing cryptic exons and TDP-43 nuclear clearance, we performed combined BaseScope-ISH and IHC on paraffin-embedded tissue sections using RNA-protein Co-detection Ancillary Kit (Advanced Cell Diagnostic Inc. Cat#323180) according to the manufacturer’s instructions. Sections were first subjected to pretreatment and then incubated overnight at 4 °C with the primary antibody, TDP-43 N-terminus (1:200, 10782-2-AP, Proteintech) diluted in the kit-supplied co-detection diluent. The following day, excess primary antibody was removed by washing with PBST and sections were post-fixed in 10% neutral buffered formalin for 30 min at room temperature. Sections were washed and proceeded with the BaseScope assay as described above. Upon completion of the detection step, sections were treated with a co-detection blocker and incubated with the appropriate secondary antibody. Protein signals were subsequently visualized using 3,3′-diaminobenzidine (DAB) staining. Counterstaining was performed with 50% Gill’s hematoxylin solution No. 1 (Electron Microscopy Sciences, Ref# 26030), followed by a brief immersion in 1 N ammonium hydroxide (Sigma-Aldrich, Cat# 320145). Sections were then dipped in fresh xylene and mounted using EcoMount (Biocare Medical, Cat# EM897). Imaging was performed using a Zeiss Apotome inverted brightfield microscope (Zeiss, Germany).

### Statistical analysis

Graphs were generated using GraphPad Prism. Individual data points are also shown. One-way analysis of variance (one-way ANOVA) for multiple comparisons was performed using the GraphPad Prism software (La Jolla, CA, USA). In all tests, values of *p* < 0.05 were considered significant. The number of biological replicates and details of statistical analyses are provided in the figure legends.

## Discussion

While tauopathies with co-pathology of TDP-43 has long been recognized as a major component of ADRD [14, 16–20, 62], the acuity of this problem was exposed in recent FDA-approved drugs targeting only β-amyloidosis [63, 64] as many participants failed to respond positively for a drug that does not address the pathophysiology of tau, α-synuclein or TDP-43. The presence of these co-pathologies is thought to drive accelerated neurodegeneration and steeper cognitive decline, which raises an important question as to how these co-pathologies contribute to disease pathogenesis. Since TDP-43 co-pathology has not only been observed with tauopathy in AD brains, but also in cases of FTLD [22], CBD [23], PART [24] and PSP [25], clarifying how this RNA binding protein influences tauopathy would provide insight into pathogenic mechanisms and novel therapeutic targets.

We previously discovered that a major function of TDP-43 is the transcriptome-wide repression of non-conserved cryptic exons, which is impaired in neurodegenerative diseases exhibiting TDP-43 pathology [26, 65]. That TDP-43 dysfunction reflects TDP-43 pathology and drives neuron loss is strengthened through the identification of disease-associated TDP-43 dependent cryptic exon targets [32–35], nuclear clearance without TDP-43 cytoplasmic aggregates in AD cases and with TDP-43 pathology [41], and the discovery that TDP-43 dysfunction occurs early in disease, including the pre-symptomatic stage [29–31]. These studies support the view that TDP-43 dysfunction occurring during the presymptomatic stage of disease may influence tauopathy to accelerate neuron loss in MEDs exhibiting the co-pathologies of tau and TDP-43. To mimic the early TDP-43 dysfunction that may occur in human disease, we employed mice lacking TDP-43 in their forebrain neurons and crossed them to a tau-seeding model to generate novel MED mouse models designed to clarify the influence of TDP-43 dysfunction on exacerbated neuron loss in human tauopathies with co-pathology of TDP-43.

Our findings are consistent with the view that TDP-43 LOF in forebrain neurons exacerbates tauopathy-dependent atrophy by sensitizing vulnerable neurons to caspase 3-dependent endoproteolysis of tau: from most vulnerable pyramidal neurons in CA2/3 to granule neurons in the DG and to the least in CA1 (Fig. 8F). The selective vulnerability of neuronal populations is dependent on their sensitivity to various risk factors; for example, in the presence of a tau seed (human hTau4R fragment), the amyloid plaque (from APP-PS1 mice) facilitated the pathological conversion of tau leading to neuronal loss primarily in CA1 and DG, but not CA2/3, neurons [46]. Note that the Tau4R tau seed alone in wild type mice has no effect on tauopathy or neuron loss, suggesting that CA1 and DG neurons are selectively vulnerable to tau seeding stimulated by the amyloid plaque [46]. In contrast, the selective vulnerability of hippocampal neurons is demonstrated by the sensitivity of CA2/3 and DG neurons to TDP-43 dysfunction-induced caspase 3-dependent tau cleavage whereas such sensitivity is blunted for those in CA1. This view is supported by our two complementary approaches, *Tau4R* transgene expression and AAV-PHP.eB-Tau4R to modestly or robustly seed tauopathy, respectively, in *CaMKIIα-Cre*^*ER*^;*Tardbp*^*f/f*^ mice. We demonstrated for the first time that in the presence of a tau seed, TDP-43 LOF can facilitate the pathological conversion of mouse endogenous tau leading to exacerbated neurodegeneration (Figs. 1 and 2), correlated with the amount of tau seeding (Figs. 3 and 4). As shown previously, the Tau4R seed by itself does not cause any pathological conversion of tau or neuronal pathology by 25 months-of-age. However, with the addition of amyloid plaque, Tau4R can facilitate the pathological conversion starting from the age of 18 months [46]. Now, we show that TDP-43 dysfunction can also facilitate this pathological conversion of tau by 25 months-of-age, and our AAV-Tau4R delivery can accelerate this conversion starting 6 months post-delivery. This data suggests that pathological conversion of tau depends on different factors including doses of Tau4R seed.

In addressing the potential mechanism underlying pathological conversion of tau triggered by TDP-43 dysfunction, we showed that higher levels of caspase 3-cleaved tau is correlated with the amount of tau seeding to exacerbate neuron loss extending beyond CA2/3 to include those of the DG and CA1 (Figs. 3 and 4), suggesting that TDP-43 LOF determines the selective vulnerability of neurons to caspase mediated endoproteolysis of tau within the mouse hippocampal circuit. Our observation that inclusion of cryptic exons precedes caspase 3-mediated endoproteolysis of tau in human iPSC-derived cortical neurons deficient in TDP-43 (Figs. 7 and 8) corroborated our observations that nuclear clearance of TDP-43 occurs in cells accumulating cleaved tau in this MED mouse model (Fig. 6). Our discoveries suggest that TDP-43 dysfunction promotes endoproteolysis of endogenous tau by caspase 3 to exacerbate tauopathy- and age-dependent neuron loss in human tauopathies with co-pathology of TDP-43.

Importantly, we also show in our MED mouse model that loss of TDP-43 leads to cryptic exon inclusion and subsequent activation of caspase 3 (Figs. 5 and 6), suggesting that TDP-43 dysfunction could be a novel therapeutic target to attenuate neurodegeneration in MEDs with co-pathologies of tau and TDP-43. Evaluating our recently validated AAV gene therapy strategy to complement TDP-43 LOF [26, 65] as a monotherapy or in combination with a tau drug, such as an ASO against tau (currently in Phase II clinical trials), using our novel *Tau4R; CaMKII-CreER; Tardbp*^*f/f*^ mouse or human model of MED should be instructive.

Based on our proposed potential mechanistic model (Fig. 8F), there are several strong predictions that would further establish the pathogenic mechanism whereby TDP-43 LOF exacerbates neurodegeneration in human tauopathies with co-pathology of TDP-43. First, our studies have not directly considered the role of β-amyloidosis in the relationship to TDP-43 LOF that occurs in cases of AD-TDP. We and others previously found that the β-amyloid plaque is necessary for the pathological conversion of tau [46, 47, 66]. That the amyloid-β plaque in the presence of TDP-43 depletion also promotes caspase 3 activation [48] would suggest the idea that β-amyloidosis might facilitate and exacerbate tauopathy in neurons lacking TDP-43 through this same mechanism, a prediction that can be addressed using mouse models of β-amyloidosis [46]. Future studies will be necessary to determine whether and how β-amyloid plaques and TDP-43 LOF converge on the caspase 3-dependent cleavage of tau to drive tauopathy.

Another strong prediction is that the inhibition of caspase 3 activation using pharmacological or genetic approaches would attenuate tauopathy in either of our mouse or human models of MED. Supporting this view are previous studies documenting: (1) inhibition of endoproteolysis of tau (at D421) using the specific antibody TauC3 (which recognizes the truncated tau neoepitope) substantially prevents the seeding competency of AD brain lysate with high molecular weight tau aggregation in cultured cells [67]; (2) overexpression of caspase 3 in neuronal cells increases tau phosphorylation and tauopathy [68, 69] (3) co-expression of humanized caspase 6 and truncated tau (TauΔD421) fails to lead to tauopathy or neurodegeneration in the aged mouse brain [70]; (4) mice expressing C-terminal truncated tau at D421 exhibit synaptic and cognitive impairments through oligomerization of tau [71]; (5) AAV delivery of C-terminal truncated tau to mouse brains results in hyperphosphorylation and oligomerization of tau leading to aggregated tau tangles [72]; and (6) caspase inhibition attenuates [73] caspase 6 cleaved tau in iPSC neurons derived from *MAPT-*V337M associated FTD.

Based on our previous finding that the central role of TDP-43 is to repress the splicing of cryptic exons [26, 65], and inclusion of cryptic exons occurs before caspase 3 activation, we hypothesize that cryptic exon inclusion could promote caspase 3-mediated endoproteolysis of tau to drive tauopathy. We speculate that inclusion of multiple cryptic exons, as opposed to any specific ones, underlies activation of caspase 3 because cryptic exons are nonconserved [26, 56], yet we observed caspase 3 cleavage of tau in both mouse and human models lacking TDP-43.

Future studies will be necessary to resolve these two possibilities. Nevertheless, our discoveries establish a novel mouse model of MED exhibiting the co-pathology of tau and TDP-43, propose a pathogenic mechanism underlying exacerbated neuron loss and offer a potential therapeutic strategy designed to either complement TDP-43 dysfunction [26, 65] and/or a combination therapy to target cryptic exon splicing and tau in vulnerable neurons of these devastating human disorders currently without effective therapy.

## Supplementary Information

Below is the link to the electronic supplementary material.

Supplementary Material 1

## Data Availability

No datasets were generated or analysed during the current study.
