# Supplementary material for: TDP-43 dysfunction facilitates the pathological conversion of tau
Source: Mol Neurodegener. 2026 Jul 4;21:58. doi: 10.1186/s13024-026-00968-8 (PMC13613616; doi:10.1186/s13024-026-00968-8)
Supplement: Supplementary file 1 [file 13024_2026_968_MOESM1_ESM.docx]

**Supplementary Figures**

**Supplementary Figure 1:** Breeding strategy for generating *CaMKII-CreER;Tardbp^f/f^*;*hTau4R* mice and littermate controls. (#) Indicates the Mendelian frequency of pups from each litter expected to carry the genotype of interest

**Supplementary Figure 2**: (**A-B)** Analysis of relative area measurements of hippocampus, cortex and cerebellum using brain sections from three levels of 19-month-old mice upon TDP-43 deletion at 12 months of age. Representative whole brain stitched images stained with cresyl violet (CV) from three different levels (from midline, commonly used 10^th^, 20^th^, and 30^th^ serial section from each group of 20- and 24-month-old mice, scale bar, 500μm). **(C)** Immunohistochemical analysis of the hippocampus in sagittal sections of brains of 25-month-old WT (n=3), Tau4R (n=4), TDP-43 cKO (n=5) and Tau4R;TDP-43cKO mice (n=4). The upper panel shows immunohistochemical staining using an antibody specific to NeuN to detect neurons, and the lower panel shows images using an antibody specific to mouse TDP-43 showing depleted TDP-43 in mouse hippocampus (scale bar, 200μm).

**Supplementary Figure 3: Pathological tau immunoreactivity as observed in different brain regions** **in Tau4R:TDP-43cKO mice:** Immunoreactivity of phosphorylated tau at pSer202, pThr205 using AT8 antibody, was observed in brain regions including entorhinal cortex (EC), cerebral cortex (CTX), hippocampus (HP); carpus callosum (CC) and thalamus (TH) of *Tau4R;TDP-43cKO* 25-month-old mice. Arrow indicates immunoreactivity in cells, Scale bar, 40μm.

**Supplementary Figure 4:** Immunofluorescence analysis corroborated the immunohistochemistry results of tau4R fragment and showed an age-dependent increase in the accumulation of hTau4R fragment using 4R repeat recognizing antibody (Tau4R;E7T4F) in subregions of HP and CTX of *Tau4R* mice (aging 15, 19 & 24 M), but not in *WT* mice right panel (24M) (Scale bar, 50 μm)

**Supplementary Figure 5.** **(A)** BaseScope analysis of whole brain sections from wild-type or TDP-43cKO mice 2 months post injection showing the presence of viral RNA in the hippocampus and region of cortex right above the hippocampus. (scale bar, 1000µm). **(B)** Representative immunohistochemical analysis and **(C)** quantification of the hippocampi of 19-month-old *Tau4R* (n=4) mice and 12-month-old WT (n=4) mice sacrificed 6 months post injection with AAV-PhP.eB-hTau4R using an antibody against Tau4R. While several hippocampal cells express Tau4R in *Tau4R* mice, Tau4R expression is greatly increased and more widespread in not only cell bodies but also axons upon AAV delivery (scale bar, 20µm) (*P=0.0286).

**Supplementary Figure 6**. **Caspase 3 is activated leading to cleavage of tau in hTau4R-expressing mice lacking TDP-43.** **(A-B)** Immunohistochemical analysis of brain sections of 19-month-old WT (n=6), Tau4R (n=6), TDP-43cKO (n=5) and Tau4R;TDP-43cKO mice (n=6) or 25-month-old WT (n=3), Tau4R (n=4), TDP-43cKO (n=5) and Tau4R;TDP-43cKO mice (n=4) in the CA1 region of the hippocampus using antibody against cleaved caspase 3 (scale bar, 50µm) **(C)** caspase-cleaved tau (TauC3) in the CA2/3 region of the hippocampus (HP) (CA2/3) and cortex (CTX) (Arrow heads indicate positive immunoreactivity of cleaved caspase 3 and cleaved tau, scale bar, 50μm).

**Supplementary Figure 7: (A)** Immunohistochemical analysis of cleaved tau (TauC3) in brain sections of 19 and 25 M old *WT* (n=3), *Tau4R* (n=4), *TDP-43*c*KO* (n=5) and *Tau4R;TDP-43cKO* mice (n=4) in the CA1 region of the hippocampus using antibody against truncated tau. The arrowheads show the cleaved tau signal in *TDP-43*c*KO* and *Tau4R;TDP-43cKO* aged (25 M), but not in WT and Tau4R transgenic mice. **(A-B)** Immunoblotting analysis of cleaved tau using TauC3 antibody, showing specific truncated tau band. **(C)** Densitometric analysis showed elevated levels of cleaved tau in Tau4R;TDP-43cKO as compared to WT and Tau4R mice (*P=0.024). (D) To testing the specificity of TauC3 immunoreactivity: Immunohistochemical analysis of cleaved tau (TauC3) in brain sections of 25 M old *Tau4R;TDP-43cKO* mice; shows immunoreactivity in regular staining with TauC3 antibody in different brain regions (Right column). The arrowheads show the cleaved tau signal. The without primary antibody (Left column) and secondary antibody (Middle column) incubation show no immunoreactivity (brown staining).

**Supplementary Figure 8.** Representative immunofluorescence images showing **(A)** TDP-43 knockdown (scale bar, 20µm) and **(B)** caspase 3 activation on day 15 in i3Neurons treated with lentivirus targeting TDP-43 (scale bar, 50µm). **(C)** Representative immunofluorescence image of colocalization of cleaved caspase 3 and cleaved tau (scale bar, 20µm)

**Supplementary Figure 9.** Nuclear TDP-43 depletion with co-occurrence of *Unc13a* cryptic RNA in TDP-43cKO mice. Co-detection of *Unc13a* cryptic exon and TDP-43 protein, respectively, using RNA *in situ* hybridization (BaseScope) and immunocytochemical analysis using antibody specific to TDP-43 in CA1 or cortex of *WT* (left panels) or *TDP-43cKO (*right panels*)* mice. Note that arrowheads point to neurons harboring *Unc13a* cryptic exon (pink dots) with nuclear clearance of TDP-43 (lacking brown stain in nucleus); scale bar, 20µm).
